# Investigating and Treating a Corneal Ulcer Due to Extensively Drug-Resistant Pseudomonas aeruginosa

**DOI:** 10.1128/aac.00277-23

**Published:** 2023-05-10

**Authors:** Morgan K. Morelli, Amy Kloosterboer, Scott A. Fulton, Jennifer Furin, Nicholas Newman, Ahmed F. Omar, Laura J. Rojas, Steven H. Marshall, Mohamad Yasmin, Robert A. Bonomo

**Affiliations:** a University Hospitals Cleveland Medical Center, Cleveland, Ohio, USA; b Case Western Reserve University School of Medicine, Cleveland, Ohio, USA; c Assiut University, Assiut, Egypt; d Louis Stokes Cleveland Department of Veterans Affairs Medical Center, Cleveland, Ohio, USA; e CWRU-Cleveland VAMC Center for Antimicrobial Resistance and Epidemiology (Case VA CARES), Cleveland, Ohio, USA

**Keywords:** antibacterial agents, cefiderocol, corneal ulcer, imipenem, keratitis, lubricant eye drops, polymyxin B, *Pseudomonas aeruginosa*

## Abstract

Resistant Gram-negative bacteria are a growing concern in the United States, leading to significant morbidity and mortality. We identified a 72-year-old female patient who presented with unilateral vision loss. She was found to have a large corneal ulcer with hypopyon. Culture of corneal scrapings grew extensively drug-resistant Pseudomonas aeruginosa. Treatment involved a combination of systemic and topical antibiotics. Whole genome sequencing revealed the presence of *bla*_VIM-80_, *bla*_GES-9_, and other resistance determinants. This distinctive organism was linked to an over-the-counter artificial tears product.

## INTRODUCTION

Resistant Gram-negative pathogens are a growing concern in the United States, leading to significant morbidity and mortality (1). When clinicians encounter such pathogens, it is essential to consider not only the complex management issues, but also the likely source of infection. On 1 February 2023, the Centers for Disease Control and Prevention (CDC) issued a Health Alert Network Health Advisory regarding an outbreak of Pseudomonas aeruginosa associated with over-the-counter (OTC) artificial tears. These infections were caused by a P. aeruginosa strain producing Verona integron-mediated metallo-β-lactamase (VIM) and Guiana extended-spectrum β-lactamase (GES) (2). This bacterial strain was extensively drug resistant (XDR), meaning that it retained susceptibility to only one or two antimicrobial categories (1). We describe a case of this same infection seen 3 months prior to the report. The patient was using the same brand of OTC artificial tears implicated in the health advisory.

## CASE PRESENTATION

In November 2022, a 72-year-old female patient presented to the emergency department with painless loss of vision in her left eye. She had a past medical history of metastatic breast cancer to the bone, including Meckel’s cave, complicated by left trigeminal neuralgia and desensitization. Consequently, she developed a left neurotrophic cornea. Per patient report, 2 weeks before presentation, she consulted an outside provider, who prescribed artificial tears, a bedtime ointment, and lifitegrast eye drops for bilateral dry eye syndrome. Approximately 1 week before admission to the hospital, she noticed decreased visual acuity and a significant change in appearance of the left eye. That morning, the patient noticed yellow discharge on her pillow and acutely decreased vision on the left. She denied a history of contact lens use, trauma to the eye, or recent exposure to pooled water.

Examination was notable for a reactive pupil of the right eye with no view of the left pupil and no relative afferent pupillary defect by reverse. The visual acuity of the left eye was light perception with poor projection and normal intraocular pressure. Slit lamp examination revealed significant conjunctival injection, along with a subtotal epithelial defect and dense corneal infiltrate associated with a 3-mm hypopyon. The corneal ulcer was cultured. She was discharged with fortified vancomycin and tobramycin ophthalmic solution drops hourly and oral doxycycline 100 mg twice daily.

**FIG 1:**
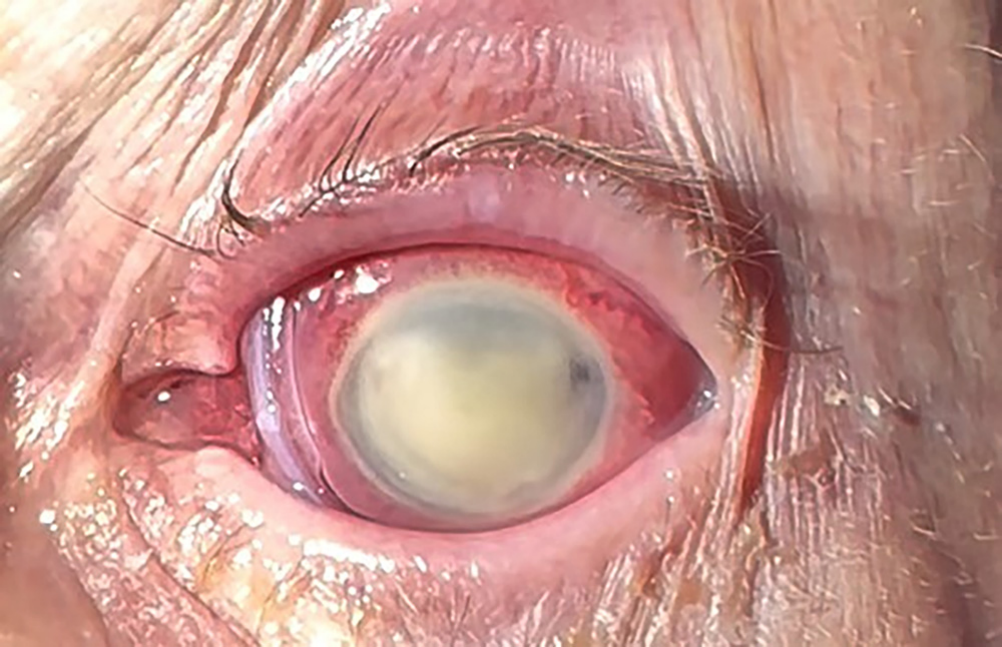
Total hypopyon with amniotic membrane in place to facilitate healing of the total epithelial defect.

She presented in follow-up to the cornea specialist the next day. The left-sided epithelial defect was now total, and the hypopyon had completely filled the anterior chamber. Preliminary culture showed growth of a multidrug-resistant (MDR) P. aeruginosa strain, with further antimicrobial susceptibility testing (AST) in progress. Given her full epithelial defect, a cryopreserved amniotic membrane was placed to facilitate healing (Fig. 1). The patient was admitted to the hospital, and the infectious diseases department was consulted. While additional AST was pending, intravenous (i.v.) ceftazidime-avibactam was initiated, and her antibiotic drops were continued. The microbiology was finalized with an XDR strain of P. aeruginosa susceptible to only cefiderocol (Table 1). AST was performed using gradient diffusion for all antibiotics except for colistin (tested by broth microdilution) and cefiderocol (tested by disk diffusion).

**TABLE 1:** Antibiotic susceptibilities of the extensively drug-resistant Pseudomonas aeruginosa isolate

Antibiotic	MIC (μg/mL)	Interpretation^*a*^
Amikacin	>32	R
Aztreonam	>16	R
Meropenem	16	R
Meropenem-vaborbactam	8	R
Imipenem	4	I
Cefepime	>16	R
Cefiderocol	23 mm	S
Ceftazidime	>256	R
Ceftazidime-avibactam	>256	R
Ceftolozane-tazobactam	>256	R
Gentamicin	>8	R
Ciprofloxacin	>2	R
Levofloxacin	>4	R
Piperacillin-tazobactam	32	I
Colistin	4	R
Tobramycin	>8	R

a I, intermediate; R, resistant; S, susceptible.

## CHALLENGE QUESTION

Based on the AST, what systemic antimicrobial therapy would you recommend in addition to topical antibiotics?

A. Cefiderocol monotherapy
B. Cefiderocol combination therapy
C. Ceftazidime-avibactam plus aztreonam
D. Imipenem-cilastatin-relebactam
E. No systemic therapy

## TREATMENT AND OUTCOME

The patient was initiated on i.v. cefiderocol 2 g every 8 h per the antibiotic susceptibilities. She was also treated with imipenem-cilastatin and polymyxin B/trimethoprim ophthalmic solutions and continued on oral doxycycline to aid in stabilization of the cornea during wound healing.

As part of the investigation into the source of this XDR infection, the patient provided the two OTC lubricant bottles she was using. An XDR P. aeruginosa isolate with the same susceptibility pattern was recovered from her OTC EzriCare artificial tears. Further testing was also done on the patient’s P. aeruginosa isolate. After *bla*_VIM_ and *bla*_GES_ were detected by PCR, whole-genome sequencing confirmed that the isolate belongs to sequence type 1203 (ST1203), possesses 7,082,165 base pairs in its genome, and harbors *bla*_VIM-80_ and *bla*_GES-9_, chromosomally located within a class 1 integron and a *Tn3* family transposon, respectively (see Fig. S1 in the supplemental material). The complete resistome is described in the supplemental material. The susceptibility results of additional antibiotics and antibiotic combinations are shown in Table S1.

Two weeks later, the patient was discharged from the hospital to complete a total 3-week course of i.v. cefiderocol. She continued the polymyxin B ophthalmic solution, as well as doxycycline. She had complete resolution of the hypopyon with improvement in the epithelial defect. Unfortunately, 2 months after her initial presentation, she developed low intraocular pressure and was found to have choroidal detachment. At the time of publication, the vision potential in the eye remains poor, given the extent of her injuries.

The emergence of MDR bacteria is one of the most concerning threats in modern medicine. These have occurred with both health care-associated and community-acquired infections (3). P. aeruginosa is known to have multiple chromosomally encoded and acquired resistance mechanisms, and the prevalence of resistant strains has been increasing. Most carbapenem-resistant P. aeruginosa strains in the United States possess non-carbapenemase-mediated mechanisms (4).

VIM-producing *Enterobacterales* strains hydrolyze many β-lactam antibiotics, including carbapenems (4, 5). Previously described infections with this strain were health care associated (6). VIM P. aeruginosa is associated with high mortality rates (7). GES is a less common extended-spectrum β-lactamase that also confers resistance to carbapenems (8). GES-producing P. aeruginosa strains have been previously reported in the United States, with various susceptibility patterns (9).

The P. aeruginosa strain identified in the CDC Health Advisory is described as the first with both VIM and GES producing carbapenem resistance in the United States (2). These isolates were only susceptible to cefiderocol, as was also discovered in our patient. In addition to the complex management of this patient, when confronted with this unique bacterium, identifying a source was paramount. Given the use of OTC ophthalmic solutions and the lack of other risk factors, our initial efforts focused on obtaining and culturing these drops. Our patient provided the bottle of artificial tears, which grew an XDR P. aeruginosa isolate with the same resistance pattern when cultured. The pharmaceutical company voluntarily recalled the implicated product swiftly after the CDC health alert was announced (10).

The primary mode of antibiotic treatment of corneal infections is topical medications in order to overcome blood-ocular barriers and the avascular nature of the cornea, which may limit systemic antibiotic exposure in the eye (11). The patient’s P. aeruginosa isolate demonstrated resistance to all commercially available ophthalmic preparations. We chose topical imipenem since the isolate exhibited intermediate susceptibility, and application of the antibiotic is directly to the site of infection. This was compounded as an ophthalmic preparation, which has been used previously for MDR bacterial keratitis (12). Polymyxin B is available commercially as part of ophthalmic preparations and may have synergy with some antibiotics, including imipenem (13). Interestingly, the colistin MIC of 4 μg/mL, now considered resistant by the Clinical and Laboratory Standards Institute, is still categorized as susceptible by the European Committee on Antimicrobial Susceptibility Testing (14, 15). There was likely bactericidal activity of polymyxin B in addition to the possible synergy with imipenem, particularly with the high concentrations achieved with topical application. Systemically, cefiderocol was the only fully susceptible option. The intraocular penetration of cefiderocol is unknown. Other cephalosporins, such as ceftazidime, have been reported to achieve therapeutic aqueous humor concentrations after i.v. administration (16). Resolution of the patient’s hypopyon supports that the patient’s antimicrobial treatment plan was successful.

The whole-genome sequence is available at NCBI GenBank under BioProject accession number PRJNA952532.
