# Supplementary material for: Investigating and Treating a Corneal Ulcer Due to Extensively Drug-Resistant Pseudomonas aeruginosa
Source: Antimicrob Agents Chemother. 2023 May 10;67(7):e00277-23. doi: 10.1128/aac.00277-23 (PMC10358754; doi:10.1128/aac.00277-23)
Supplement: Supplemental file 1 — Supplemental material. Download aac.00277-23-s0001.docx, DOCX file, 0.2 MB [file aac.00277-23-s0001.docx]

Appendix:

Upon reception at the research lab, antimicrobial susceptibility testing (AST) was performed using reference broth microdilution (BMD), with the exception polymyxin B which was assessed by broth macro dilution according to the Clinical and Laboratory Standards Institute guidelines (CLSI; M100-Ed32). Iron-depleted Cation adjusted Mueller Hinton media was used for all cefiderocol testing. The Kirby-Bauer disk diffusion method was used to assess synergy against various antibiotic combinations. The antibiotics selected for combination testing included aztreonam (ATM; 30 μg), imipenem (IMI; 10 µg), avibactam (AVI; 20 μg), cefiderocol (FDC, 30 μg), polymyxin B (POLB, 300 ug), and ceftazidime-avibactam (CZA, 30/20 µg) (Table S1).

*bla* genes were initially detected using the Streck ARM-D, β-lactamase kit (Cat No. 250045) and Streck ARM-D TEM/SHV/GES kit (Cat No. 250054) directly from isolated colonies, following the manufacturer’s instructions. Subsequently, DNA was extracted using the MasterPure Gram Positive DNA purification kit following the manufacturer’s instructions (Epicentre, Madison, WI). Libraries were prepared using the Oxford Nanopore Technology’s Rapid Barcoding Kit and whole genome sequencing was performed in a ONT MinION. The genome was de novo assembled with Flye, polished with Medaka and annotated using BV-BRC (Bacterial and viral bioinformatics resource center) and confirmed with BLAST (Basic Local Alignment Search Tool, NCBI). Resistome and MLST were determined using ResFinder 4.1 and MLST 2.0, respectively from the Center for Genomic Epidemiology (https://www.genomicepidemiology.org/services/).

The assembly resulted in a single, with a total length of 7,079,333 bp, an average G+C content of 65.86%, and 6,817 protein coding sequences (CDS) (Figure S1). Resistome analysis showed this isolate carried several chromosomally encoded antibiotic determinants including *aph*(3')-IIb, *rmt*F, *aadA*1, *aph*(3'')-Ib, *aph*(6)-Id (aminoglycosides); *bla* _PDC-19a_, *bla*_GES-9_, *bla*_VIM-80_, *bla*_OXA-10_, *bla*_OXA-395_ (beta-lactams); *qacE* (quaternary ammonium); *aac*(6')-Ib-cr (Fluoroquinolone and aminoglycosides); *fosA* (fosfomycin) ; *erm(A)* (macrolide); *catB7*, *floR* (phenicols); *sul1* (sulfonamides); *tet(G)* (tetracycline); *dfrA5* (trimethoprim). Further analyses showed that the genes encoding PBP3, OprD, and MexA did not have any mutations as compared to PAO1, whereas mutations were present in MexB (I186V, S1041E, V1042A), MexR (V126E, P143L), GyrA (T83S dek 909-910) and ParC (S87L and T556S). Additionally VIM-80 Metallo-β-lactamase was shown to be integron mediated (*bla*_VIM-80_ immediately after *intI1*), whereas GES-9 β-lactamase is within a *Tn3* family transposon as shown in Figure S1 A and B, respectively.

Table S1. Additional antibiotic susceptibilities performed at a research laboratory

| **Antibiotic(s)** | **MIC (µg/mL)** | **Disc (mm)** |
| --- | --- | --- |
| Polymyxin B macro | >8 | 11 |
| Imipenem | 16 | 14 |
| Aztreonam | 64 | NA |
| Aztreonam/avibactam | 4 | NA |
| Cefiderocol | 1 | 26 |
| Cefiderocol/aztreonam | 0.5 | NA |
| Ceftazidime-avibactam | 64 | 12 |
| Cefiderocol/polymixin B | NA | 24 |
| Cefiderocol/imipenem | NA | 28 |
| Cefiderocol/polymixin B/imipenem | NA | 30 |

MIC: minimum inhibitory concentration, NA: not applicable


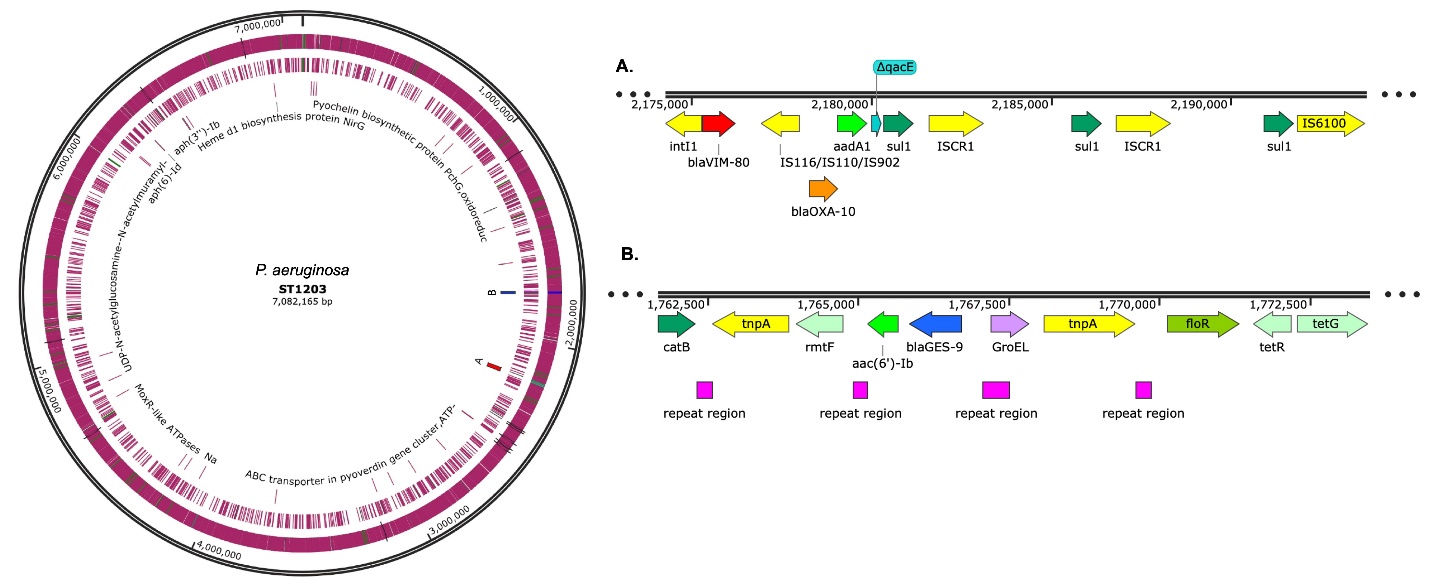


Figure S1. Chromosomal map of the *Pseudomonas aeruginosa* strain. A. Genetic environment of VIM-80; B. Genetic environment of GES-9.
